# Self-healing Characteristics of Collagen Coatings with Respect to Surface Abrasion

**DOI:** 10.1038/srep20563

**Published:** 2016-03-24

**Authors:** Chang-Lae Kim, Dae-Eun Kim

**Affiliations:** 1 Center for Nano-Wear, School of Mechanical Engineering, Yonsei University, Seoul, 03722, Republic of Korea

## Abstract

A coating based on collagen with self-healing properties was developed for applications in mechanical components that are prone to abrasion due to contact with a counter surface. The inherent swelling behavior of collagen in water was exploited as the fundamental mechanism behind self-healing of a wear scar formed on the surface. The effects of freeze-drying process and water treatment of the collagen coatings on their mechanical and self-healing properties were analyzed. Water was also used as the medium to trigger the self-healing effect of the collagen coatings after the wear test. It was found that collagen coatings without freeze-drying did not demonstrate any self-healing effect whereas the coatings treated by freeze-drying process showed remarkable self-healing effect. Overall, collagen coatings that were freeze-dried and water treated showed the best friction and self-healing properties. Repeated self-healing ability of these coatings with respect to wear scar was also demonstrated. It was also confirmed that the self-healing property of the collagen coating was effective over a relatively wide range of temperature.

Self-healing may be defined as the ability of a material to restore its original physical state after being altered by mechanical stress or chemical reaction^1^. It is a highly desirable property for a material that is prone to mechanical damage such as abrasion or scratching. In this regard, numerous works on the self-healing behaviors of various types of materials have been performed with the motivation to develop highly durable and replacement-free components. Use of a microencapsulated healing agent to heal cracks formed in structural composite was proposed by White *et al*.^2^. However, there was a limitation that the microcapsule could only be used once. Toohey *et al*. suggested another method for healing ruptures by using microvascular materials in order to solve the limitations of the microencapsulated healing method^3^. Another method for repeated self-healing effect was proposed by Li *et al*. in which composite sandwich structures made of a shape memory polymer with a foam core was utilized^4^.

The self-healing methods described above generally require the use of a healing agent that functions through some sort of a chemical reaction. However, in situations where chemicals cannot be readily applied due to concerns about contamination and biocompatibility, other self-healing methods need to be utilized. In this regard, materials such as collagens that are known to have autonomous self-healing ability would be beneficial^5^,^6^. In general, collagens have been extensively used in the medical field for several purposes due to its biocompatibility, safety and biodegradability^7^,^8^. Collagens are also well known to swell in high humidity environment or upon contact with water^9^,^10^. Regarding the swelling behavior of collagens, many studies have been conducted to understand the mechanism and the physical properties of these materials. For instance, the effects of collagen fiber density on the mechanical properties and swelling behavior of collagens have been reported^11^,^12^.

Considering the advantages of collagens such as self-healing property and biocompatibility, there is a great potential for these materials to be utilized in a wider range of applications. Particularly, the self-healing properties may be exploited to develop smart biocompatible coatings for surface protection of devices. In this regard, the durability and reliability of collagens in the form of a coating should be assessed and methods to improve these qualities need to be developed. There have been a lot of studies regarding the damage characteristics of bulk collagens for applications in bioengineering such as implant, cell culture and drug delivery^13^,^14^,^15^,^16^,^17^. On the other hand, fundamental studies regarding the mechanical properties of collagens in the form of coatings have not been explored sufficiently. Furthermore, the self-healing property of collagens is expected to offer benefits that cannot be attained with other types of coatings for surface protection applications.

In this study, collagen in the form of a coating was fabricated on glass with the aim to develop biocompatible coatings that possess self-healing ability with respect to surface damage caused by abrasion. Collagens are known to have relatively poor mechanical strength compared to other coatings used in surface protection applications^18^,^19^,^20^. Therefore, means to improve the durability of collagen coatings should be developed as well in order to effectively exploit the self-healing property of collagens. For this purpose, the physical, chemical, and mechanical properties of collagen coatings were assessed. Friction and wear tests were also performed on the collagen coatings to understand their abrasion characteristics. The self-healing effect of the collagen coating due to contact with water was determined with respect to the wear scar formed on the surface. The repeatability of the self-healing effect of the collagen coating was also assessed.

## Results

### Structure and properties of coatings

**Figure 1:**
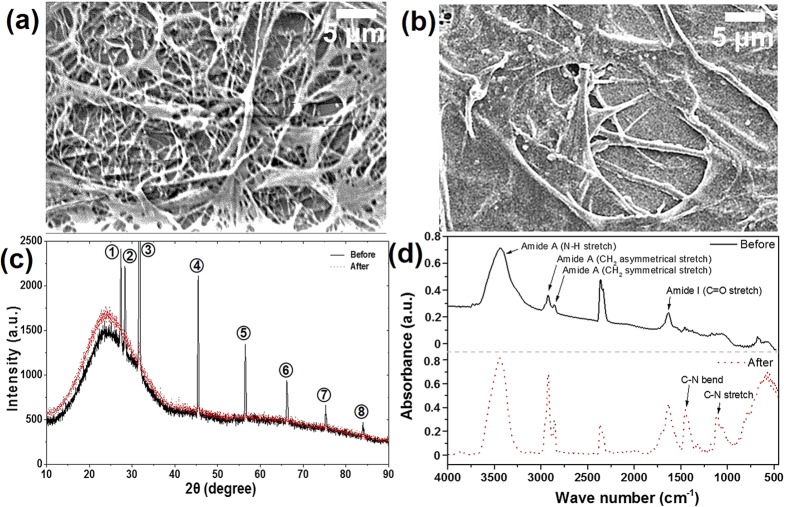
Structure and properties of collagen coatings. (**a,b**) SEM images of collagen coating specimen surface morphology after freeze-drying: (**a**) before and (**b**) after water treatment. (**c**) XRD data of collagen coating specimen before and after water treatment: Several peaks with high intensity were detected on the collagen coating before water treatment (black solid line). There was no peak with high intensity on the collagen coating after water treatment (red dash line). (**d**) FT-IR spectra for collagen coating specimens before (black solid line) and after (red dash line) water treatment.

The surface morphology of the collagen coating that was completely dried by the freeze-drying process was observed by a scanning electron microscopy (SEM, JSM-6610, JEOL Inc.) as shown in Fig. 1a. The coating had a randomly entangled porous structure of collagen fibers similar to the morphology of collagens reported previously^21^,^22^. The diameter of the collagen fibers varied from several tens to hundreds of nanometers. It was also determined that on the average, the thickness of the collagen coating was ~30 μm. The surface morphology of the collagen coating after the water treatment process was also analyzed. The collagen coating that was completely dried by the freeze-drying process was hydrated by water treatment. Then, the hydrated collagen coating was naturally dried in ambient environment to form a different collagen structure. As shown in Fig. 1b, it was evident that the surface morphology of the collagen coating changed drastically due to water treatment. Though there were still some entangled fibers the size and shape of the fibers were altered significantly. Also, on the average, the thickness of the coating was reduced significantly to ~6 μm and the average surface roughness decreased from about 2.4 μm to 0.2 μm after water treatment.

The crystallinity of the collagen coating specimens was analyzed using an X-ray diffraction (XRD, Rigaku SmartLab) method. It was found that the crystallinity of the collagen coating was also varied by water treatment as shown in Fig. 1c. For both coatings, the typical broad diffraction pattern for collagen at 2θ = 24° could be found. Also, in the case of the collagen coating before water treatment, several peaks with relatively high intensities appeared in the XRD data. From the positions of these peaks, the grain size of the crystals could be estimated using Equation (1). As shown in Table 1, the grain size of the freeze-dried collagen coating crystals was about 87 to 111 nm. On the contrary, there was no peak with high intensity in the XRD data of the collagen coating after water treatment.

The chemical state of the collagen coating specimens was analyzed by a fourier transform infrared (FT-IR) spectrometer (Vertex 70, Bruker Inc.). The FT-IR spectra for the collagen coatings before and after water treatment are shown in Fig. 1d. The typical bands such as N-H stretching, C=O stretching and CH_2_ asymmetrical/symmetrical stretching for the amide A and the amide I were observed at similar positions on both coatings. However, a few bands such as C=O stretching, C-N bending and C-N stretching appeared distinctly after water treatment. Also, compared to the completely dried collagen coating, a frequency shift in the amide I (C=O stretch) peak occurred after water treatment from 1674 cm^−1^ to 1668 cm^−1^. These results were attributed to the C=O and C-N polar groups in collagen that interacted with water molecules^23^. Thus, it was thought that hydration of the peptide groups resulted in the frequency shift, and the variation of the intensities of the amide I band indicated that the orientation of the collagen molecules was altered^24^.

Indentation tests were performed to assess the mechanical properties of the collagen coating specimens. From the indentation test results of the specimens before and after water treatment, its effects on the mechanical properties were assessed as shown in Table 2. Overall, the hardness, elastic modulus and stiffness of the collagen coating increased significantly due to water treatment. Such a change in the mechanical property of hydroxyapatite/collagen nanocomposite material was also previously reported by Chang *et al*.^25^.

### Friction and wear characteristics of collagen coatings

**Figure 2:**
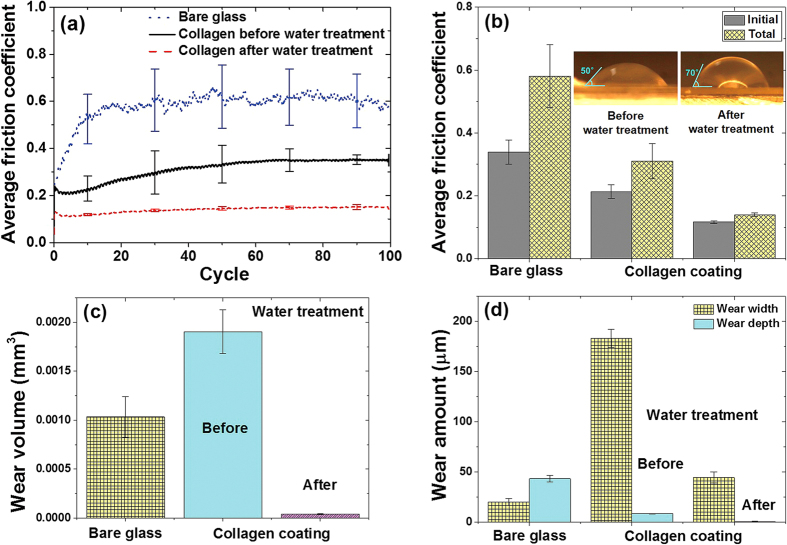
Friction and wear characteristics of collagen coatings. Average friction coefficient of glass and collagen coating specimens before and after water treatment with respect to (**a**) number of sliding cycles and (**b**) initial and total stages of sliding test. The inset optical images show the water contact angles for the collagen coating before and after water treatment. (**c**) Average wear volume and (**d**) average wear width and depth of the glass and collagen coating specimens before and after water treatment after 100 cycles of sliding.

To evaluate the friction and wear characteristics of the collagen coating specimens, sliding tests were performed using a reciprocating type of a tribotester. Figure 2a shows the average friction coefficient with respect to the sliding cycles for the collagen specimens, including glass. Glass was tested as a reference material since it was used as the substrate material for the collagen coating specimens. As shown in Fig. 2, friction coefficient of the bare glass specimen was the largest with a significant deviation. Also, the friction coefficient increased rapidly over about ten sliding cycles from a relatively low value of ~0.25 to ~0.6. This kind of frictional behavior is commonly found in metallic and ceramic sliding systems, and the cause could be attributed to increase in the plowing component of friction as wear particles are generated at the sliding interface^26^. For the collagen coating specimens, the average friction coefficient as well as the standard deviation was significantly lower compared with those of the glass specimen. The collagen coating specimen before water treatment also showed that the friction coefficient increased from a relatively low value to a higher value with increasing sliding cycles. However, this increase in the friction coefficient with respect to sliding cycles was not attributed to increase in the plowing action since a significant amount of wear particle generation was not found in the case of the collagen coating specimens. Instead, it was attributed to the increase in the contact area as the collagen coating got compressed as it experienced repeated sliding against the zirconia ball. In the case of the water treated collagen coating specimen, the friction coefficient was drastically lower and more stable than that of the untreated collagen coating specimen.

In order to assess the contribution of adhesion to the friction force, the surface energy of the collagen coatings before and after water treatment was assessed by measuring the water contact angle. As shown in the inset images of Fig. 2b, the water contact angles of the collagen coatings before and after water treatment were 50° and 70°, respectively. This result indicated that the surface energy of the collagen coating before water treatment was slightly higher than that of the collagen coating after water treatment. However, it should be mentioned that adhesion was not expected to the main reason for the large difference in the friction coefficients observed between the water treated and untreated collagen coatings. Other factors related to the enhanced mechanical properties of the water treated collagen coating were considered to be more important.

The frictional behavior of the specimens was further analyzed by observing the variation in friction coefficient with respect to sliding cycles. Figure 2b shows the average friction coefficient for the initial and total stages for the coated specimens obtained from the data in Fig. 2a. The initial stage (5 cycles) represented the frictional behavior of the coating at the onset of sliding prior to any significant alteration in the surface state of the specimen. The total stage represented the entire duration of the sliding test (100 cycles). The average friction coefficients of bare glass and collagen coatings before and after water treatment during the initial stage were 0.34, 0.21 and 0.12, respectively. Over the total sliding cycles, the friction coefficient of the specimens increased respectively to 0.58, 0.31, and 0.14.

The wear characteristics of the specimens were compared with respect to the wear volume and wear scar dimensions after 100 cycles of sliding test. It was clearly found that glass and collagen coating specimens before water treatment suffered a significant amount of wear. On the other hand, only a slight amount of wear was observed on the collagen coating specimen after water treatment. The amount of wear was quantified from the 2D profile of the wear scar. The average cross sectional wear area was calculated by measuring the grooved area below the horizontal line of the specimen surface roughness at several locations along the wear scar. Then, the total wear volume was obtained by multiplying the average wear area with the sliding stroke. Figure 2c shows the wear volume results for all the specimens. The wear volume of the collagen coating specimen before water treatment was the largest followed by glass and the coating specimen after water treatment. Particularly, the wear of the collagen coating could be reduced by about 50 times by the water treatment process. This was a remarkable improvement in the wear resistance of the collagen coating.

As for the wear of the ball, it was found that the ball slid against the glass specimen suffered the largest degree of wear compared with the other specimens. In the case of the collagen coating specimen before water treatment, some evidence of material transferred from the collagen specimen could be found. However, the ball that was slid against the collagen coating specimen after water treatment was relatively clean and intact. These results demonstrated that the overall wear characteristics of the freeze-dried collagen coating could be significantly improved by the water treatment process.

### Self-healing property of collagen coating

Following the friction and wear characterization of the collagen specimens, the self-healing property of the specimen was investigated. This was performed by comparing the wear scar formed on the collagen coating before and after self-healing. As mentioned previously, the basic mechanism behind the self-healing effect of collagen was swelling due to contact with water. Thus, water was used as a medium to heal the wear scar formed on the collagen surface. Initially, the self-healing effect was examined using the collagen coating that was not processed by freeze-drying. In this case, the wear scar formed on the collagen surface could not be recovered and no self-healing effect could be observed. Therefore, subsequent self-healing experiments were all performed using the freeze-dried collagen coating specimens.

**Figure 3:**
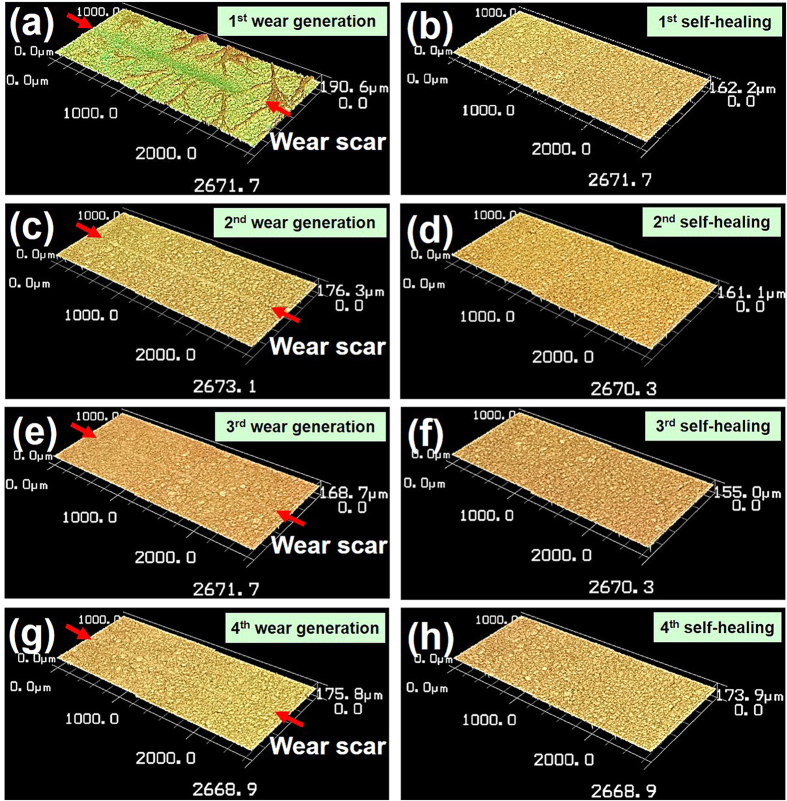
Confocal microscope images of freeze-dried collagen coating specimen with repeatable self-healing property with respect to a wear scar formed on the surface. (**a**) 1st wear scar, (**b**) after self-healing of the 1st wear scar, (**c**) 2nd wear scar, (**d**) after self-healing of the 2nd wear scar, (**e**) 3rd wear scar, (**f**) after self-healing of the 3rd wear scar, (**g**) 4th wear scar, (**h**) after self-healing of the 4th wear scar. Water was used as a self-healing medium. Red arrows indicate the two ends of the wear scar formed on the specimen surface.

The self-healing effect of the freeze-dried collagen coating with respect to the wear scar formed on the surface was clearly observed. The wear scar imparted on the surface of the collagen specimen disappeared almost instantly when a certain amount of DI water was dropped on the damaged area. Figure 3a,b show the 3D laser scanning confocal microscope (VK-X200, KEYENCE Co.) images of the collagen specimen before and after self-healing, respectively. As can be seen, the wear scar formed on the surface (Fig. 3a) could not be found after the self-healing process (Fig. 3b). Also, the surface morphology of the freeze-dried collagen coating improved with respect to uniformity and roughness as a result of contact with water as noted before. Essentially, the compressed fibers of the collagen coating due to abrasion recovered to their original state after being in contact with water. Thus, the swelling behavior of collagen was effective in producing the self-healing effect of the collagen coating with respect to a wear scar.

After confirming the self-healing property of the freeze-dried collagen coating, further tests were performed to investigate the possibility of repeated self-healing effect. The specimen with a wear scar that was self-healed by contact with water was naturally dried. Then, the wear test was performed on the same specimen to create a new wear scar as before. The new wear scar formed on the specimen after the first water treatment was significantly smaller than the first wear scar since the mechanical properties of the collagen coating improved after contact with water. It was found that the new wear scar could also be removed by the self-healing process as shown in Fig. 3c,d. Moreover, several repeated self-healing tests were all successful in removing the wear scar formed on the collagen coating surface. Figure 3 shows the self-healing test results up to 4 repeated trials. These results indicated that collagen coating had the ability to be self-healed repeatedly with respect to damage caused by surface abrasion.

**Figure 4:**
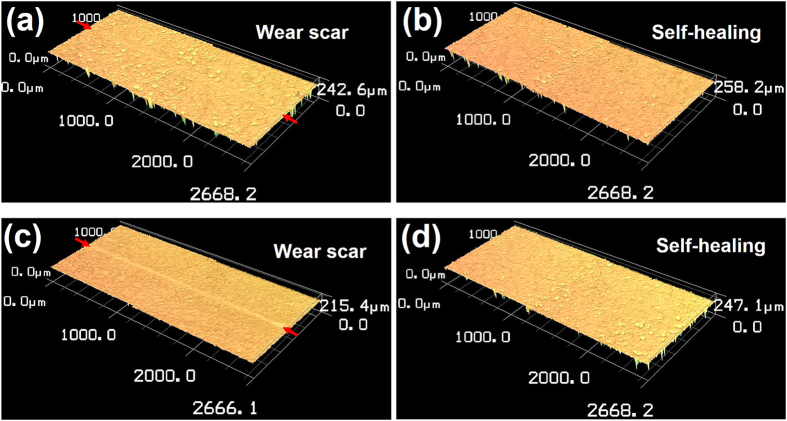
Confocal microscope images of collagen coating specimens tested under high (50 °C) and low (2 °C) temperatures. (**a**) Wear scar formed at 50 °C, (**b**) after self-healing at 50 °C, (**c**) wear scar formed at 2 °C and (**d**) after self-healing at 2 °C.

In order to assess the effect of environmental temperature on the self-healing behavior of the collagen coating, additional wear and healing tests were performed at 50 °C and 2 °C. For the 50 °C test, the collagen coating specimen was placed inside an oven and the wear test was performed under an applied load of 30 mN for 100 cycles using a simplified reciprocating tribotester that could fit inside the oven. After the wear scar was generated, it was treated with water at the same temperature to assess the self-healing effect. For the 2 °C test, the wear and healing tests were performed inside a refrigerator. Figure 4 shows the wear scar before and after the healing process for both temperatures. As can be seen from the figure, the wear scars were effectively healed even under these temperatures. Thus, it was confirmed that the self-healing property of the collagen coating was effective over a relatively wide range of temperature.

## Discussion

The present study suggests that a collagen coating with a self-healing ability may be used as a biocompatible coating for applications in mechanical components that are prone to be damaged by contact with a counter surface. The freeze-drying and water treatment processes were conducted to demonstrate the effect of self-healing as well as to improve the durability of collagen coatings that are known to have relatively weak mechanical properties. It was confirmed through the SEM analysis (Fig. 1a,b) that the surface morphology of the collagen coating was significantly varied after water treatment. Such drastic changes in the surface morphology of the collagen coating were due to reaction of the collagen fibers that are known to be sensitive to water^27^.

While the XRD data showed that the freeze-dried collagen coating was crystalline, in the case of the collagen coating after water treatment, there was no peak with high intensity in the XRD data as shown in Fig. 1c. This suggested that the crystallinity of collagen coating mostly disappeared and became an amorphous structure after water treatment. It was presumed that the physio-chemical changes incurred by the collagen coating due to water treatment led to the transformation of the collagen coating structure from crystalline to amorphous. However, identification of the exact mechanism behind this structural change was beyond the scope of this work.

From the results of the FT-IR analysis of the collagen coatings shown in Fig. 1d it was presumed that the orientation of the collagen molecules varied due to water treatment. In addition, the triple helix conformation in the structure of the collagen coating could be stabilized by the water molecules^25^. Water treatment effectively converted the triple helix into a highly hydrated state which stabilized the collagen structure with the formation of hydrogen bonds^28^. The decrease in the vibration frequency of the carbonyl group suggested that the strength of the hydrogen bond composed of the hydrogen atom and the carboxyl oxygen molecule increased. Meanwhile, three polypeptide strands with stable intramolecular bonds were present in an individual collagen molecule and high strength of collagen fibers were due to the strong intermolecular covalent bonds between the collagen molecules.

The mechanical properties of the collagen coating specimen was enhanced after water treatment. Variation in the surface and mechanical properties of the collagen coatings after water treatment was expected since the morphology and structure of the coating was varied significantly. Firstly, the surface roughness of the collagen coating decreased significantly from 2.4 to 0.2 μm. Furthermore, the thickness of collagen coating decreased about 5 times from about 30 to 6 μm. As the thickness was reduced the surface roughness also improved since the collagen fibers on the surface were compacted during the drying process. Thus, a relatively smooth and compact structure with a higher density was formed after the water treatment process. The reduction of coating thickness was due to the capillary pressure of water formed in the pores of collagen fibers during the drying process which led to the compaction of the flexible fibrous structure^29^,^30^. Essentially, the surface tension and capillary force between the collagen fibers increased the surfaces forces between the fibers. Thus, the mechanical properties of the collagen coating improved after water treatment because the sparsely entangled collagen fibers before water treatment were compactly compressed after contacting with water.

The friction coefficient of the collagen coating after water treatment was lower than that of the collagen coating before water treatment as shown in Fig. 2a,b. The reason for the improved frictional behavior of the collagen coating specimen after water treatment was attributed to its relatively low surface roughness and improved mechanical properties compared to the untreated collagen coating specimen. From these results, it was concluded that water treatment of the freeze-dried collagen coating was very effective in improving the frictional characteristics of the coating.

In the case of wear volume, the collagen coating after water treatment showed much less wear compared to the untreated collagen coating as shown in Fig. 2c. The wear characteristics of the specimens were further analyzed by comparing the wear scar width and depth as shown in Fig. 2d. A clear difference in the wear trend between the glass and the collagen coating specimens could be found. In the case of the collagen specimens, the wear scar showed significantly larger widths compared to the depths. On the other hand, the depth of the wear scar formed on the glass specimen was much higher than the width. Therefore, even though the total wear volume of the collagen coating specimen before water treatment was higher than that of the glass specimen, the wear depth was much lower. This outcome was due to the relatively low stiffness of the collagen coating which allowed for higher elastic strain in the vertical direction under a given applied load. As a result, the applied load was distributed over a larger contact area between the ball and the specimen surface which led to a large wear width. Nevertheless, the depth of wear was significantly less than the width, which was a less critical situation than the case of a relatively large wear depth for a given total wear volume. Such a wear characteristic was attributed to the mesh structure of the collagen coating which was beneficial to distribute the contact load over a large area and decrease wear in the depth direction. This concept was previously demonstrated with a carbon nanotube coating with a mesh structure fabricated on silicon^31^.

**Figure 5:**
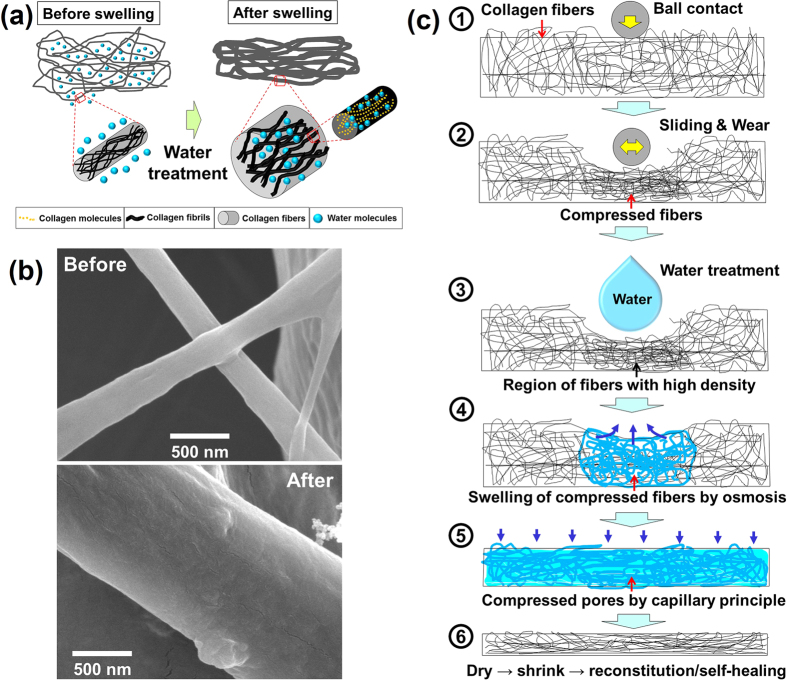
Swelling and self-healing of collagen. (**a**) Schematic of the swelling process of collagen structure consisting of fibers and fibrils. (**b**) SEM images of the collagen fiber before and after swelling due to water. (**c**) Schematic of the self-healing mechanism of a freeze-dried collagen coating with respect to a wear scar formed on the surface. ① State of ball sliding against the collagen coating; ② Wear scar formed on the surface in the form of fiber compression; ③ Water dropped on the wear scar region; ⑤ Water introduced to the wear scar region causes self-healing due to fiber swelling effect; ⑥ Water spread to the surrounding region causes contraction of the coating due to capillary effect; ⑦ After drying the coating becomes thinner and denser with improved mechanical properties.

It was confirmed that the wear scar formed on the collagen coating completely disappeared when it contacted with water. This self-healing process could be repeated for several times. After confirming the repeatable self-healing property of the collagen coating, the fundamental mechanism behind the wear scar recovery phenomenon was further analyzed. Firstly, Fig. 5a illustrates the basic swelling behavior of the collagen coating upon contact with water. The collagen structure is represented by the entangled fibers that are composed of tiny fibrils made of collagen molecules. When water is introduced to the collagen in the freeze-dried state, the water molecules penetrate into the pores of the structure as well as into the fibrils. The water molecules cause the distance between the fibrils to expand, thus enlarging the size of the collagen fibers. The detailed mechanism behind this phenomenon has been already described in previous works^32^,^33^. Figure 5b shows the SEM images of the collagen fiber before and after swelling due to water. Before swelling, the collagen fiber is in the state of complete dehydration as a result of freeze-drying. After hydration, the fiber is significantly enlarged. It is evident that diameter has increased by about 5 times.

As for the mechanism of wear scar recovery upon contact with water, the wear characteristics of the collagen coating need to be understood in conjunction to its swelling behavior. The premise that no or very little material removal occurs due to the abrasion action should be satisfied to attain the self-healing effect using the collagen coating. In other words, surface damage caused by abrasion should occur mostly in the form of compression rather than material removal. The mesh structure of the collagen coating allows for this type of surface damage to occur when a contact stress is applied to the surface repeatedly. Thus, the wear scar formed on the surface during the wear test was in the form of compressed fibers rather than detached fibers. As mentioned before, the flexible mesh structure of the collagen coating is ideal in this respect since the applied load can be effectively distributed in the plane direction, thus preventing further penetration of the counter surface in the depth direction.

The mechanism of the self-healing process of freeze-dried collagen coating with respect to a wear scar is illustrated in Fig. 5c. With introduction of water to the wear scar the compressed fibers expand by the swelling action through the osmosis process^32^. The helical peptide chains in collagen fibrils are known to be hydrophilic. Moreover, a number of carboxyl and amino groups in collagen peptides have fixed charges connected with the monovalent counter ions. Thus, when the collagen fiber comes into contact with water molecules, it undergoes an osmotic swelling process due to the Gibbs-Donnan effect^34^. Essentially, the collagen fibers have a good affinity with water and the characteristic to be swollen when water is absorbed^32^,^34^. Expansion of the fibers continues until the osmotic forces are offset by the mechanical reaction forces that arise from the elastic deformation of the fiber structure^35^. Thus, when the osmotic and elastic forces are balanced, the swelling action seizes to continue. It should be mentioned that the is not effective if the collagen coating is completed penetrated by abrasion. This is because the fibers need to be connected to attain good swelling properties, which is essential for self-healing.

While the compressed fibers of the wear scar expand to recover its original state upon contact with water, the surrounding areas of the collagen coating that come into contact with water contract due to the capillary effect. Thus, water introduced to the wear scar causes the compressed region to swell and the surrounding regions to get thinner due to the capillary effect. With repeated abrasion on the surface of the collagen coating that has already been self-healed once, the wear scar is not as deep since the mechanical properties of the coating improve due to contact with water. Nevertheless, the process described above is repeated at a smaller scale, thus allowing the collagen coating to possess the with respect to abrasion.

In conclusion, collagen in the form of a coating was successfully fabricated on glass through a relatively simple process. The structural and mechanical properties of the coatings were assessed using various analytical tools. It was found that the mechanical properties of the collagen coatings could be significantly enhanced by treating the coating with water. Furthermore, freeze-dried collagen coating demonstrated the with respect to a wear scar formed on the surface. The self-healing effect was attributed to the characteristics of the wear scar, which was formed as a compressed layer of collagen fibers, in conjunction with the swelling behavior of the fibers in water. The repeatable self-healing ability of the collagen coatings was also demonstrated. Furthermore, it was confirmed that the self-healing effect of collagen coating was effective over a relatively wide range of temperature. This type of coating may be utilized for applications in devices that are subject to surface abrasion.

## Methods

### Preparation of collagen coating

Collagens extracted from rat tails were used for fabrication of the collagen coating on glass. Protein solution (Collagen Type I, Corning^®^) with 3.44 mg/mL of concentration containing rat tail tendon was used as the main ingredient. This solution was treated with acetic acid in order to preserve the collagen molecules in the inactive state. Thus, to make the collagen solution from which collagen fibers were formed, the acidic protein solution was neutralized by mixing with phosphate buffered saline (PBS) solution, sodium hydroxide (NaOH) solution and deionized (DI) water. It has been reported that density of collagen may be controlled by the concentration of collagen solution^36^,^37^. For the purpose of this work, a collagen solution with a concentration of 2.0 mg/mL was employed. Thus, to formulate 1000 μL of collagen solution, 581.4 μL of protein solution with 3.44 mg/mL of concentration, 100 μL of PBS solution, 11.4 μL of 1 M NaOH solution and 307.2 μL of DI water were compounded.

In order to increase the adhesion of the collagen coating, the glass substrate was thoroughly cleaned and pretreated to be hydrophilic based on the methods reported in other works^38^,^39^. First, the glass substrate was cleaned by sonication in acetone for 5 minutes and again in ethanol for another 5 minutes. Finally, all the residues left on the glass surface were eliminated by sonication in DI water and dried with nitrogen gas. After the cleaning process, the glass substrate was dipped in 3-Aminopropyl triethoxysilane, silane, and glutaraldehyde solutions in sequence to increase the hydrophilicity of the glass surface. The last process was to eliminate the impurities by cleaning the glass substrate using DI water. Following the pretreatment process, a small amount of the collagen solution was dropped on the glass surface and squeezed by another piece of glass without any surface treatment to form a thin collagen film. After about 15 minutes of solidification process the top glass was carefully removed. Through this process, a thin gel type of a collagen coating that was almost transparent could be fabricated on the pretreated glass substrate.

For the freeze-drying process, the collagen gel coating was dried in a freeze-drying machine (EYELA, FDU-2110, Tokyo Rikakikai Co., LTD) for 24 hours at a temperature of −80 °C and a pressure of 7 ~ 8 Pa. Through this process the frozen water content in the collagen gel coating could be transformed directly to vapor from the solid phase, thereby preserving the integrity of the collagen fibers during the drying process. It should be mentioned that freeze-drying process is typically used to fabricate a porous scaffold structure in collagens for tissue engineering^40^,^41^,^42^. The principle of freeze-drying is based on sublimation process in which the frozen water in the collagen gel coating transforms directly to vapor from a solid phase^43^. This can be accomplished by lowering the partial pressure of water vapor after all the liquid in the coating has been frozen. After the freeze-drying process, the collagen gel transformed to a fibrous coating structure with voids that were previously filled with frozen water.

### Analyses of collagen coating properties

The surface structures of collagen coatings were analyzed using the SEM. The thickness of collagen coating was measured by the SEM observation of the cross section of the coating. Furthermore, the surface roughness of collagen coating was measured by a 3D profiler (Dektak XT, Bruker Inc.). The XRD method was used to analyze the crystal structure of the collagen coatings. The grain size (D) was calculated by Equation (1), where λ is the wavelength (0.154 nm, Cu_kα1_) of the X-ray, β_L_ is the full width at half maximum (FWHM) and θ is the position of the diffraction peak. The conformational variation of the structure of collagen coating according to water treatment was assessed using the FT-IR spectrometer with KBr beamsplitter in the range of 4000 ~ 400 cm^−1^.


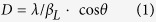


Water contact angle measurements were performed to assess the surface energies of the collagen coatings. 1 μL of deionized water was dropped on the collagen coating specimens before and after water treatment using a micrometric pipette (Research plus, Eppendorf, Germany). Then, the contact angle was measured at room temperature by using a camera. Through the measurement of the contact angles, the relative surface energies of the collagen coating specimens could be assessed.

To investigate the mechanical properties of the collagen coatings, the indentation measurement technique was used. The mechanical measurements were performed before and after water treatment process. A stainless steel ball (SUS 304) with a diameter of 1 mm was used as the indenter tip. The tip was attached to a load cell installed on a motorized linear stage that was used to move the tip in the vertical direction. The motorized linear stage had a resolution of a few nanometers and it was operated at a relatively low speed of several μm/s. The indentation measurement conditions are given in Table 3. The indentation force with respect to depth was obtained to assess the mechanical response of the collagen coating specimens. The mechanical properties such as hardness, elastic modulus and stiffness were calculated from the indentation measurement data^44^,^45^.

### Friction and wear experimental method

The friction and wear characteristics of the collagen coating specimens were investigated by using a reciprocating type of a tribotester. As shown in Table 3, the sliding stroke and speed were set to be 2 mm and 4 mm/s (1 Hz), respectively. A zirconia (ZrO_2_) ball with a diameter of 1 mm was used as the counter surface to slide against the collagen coatings. Every experiment was conducted with the new ball under an applied normal load of 9.81 mN. The sliding experiment was performed up to 100 cycles (400 mm). All the experiments were conducted in a Class 100 clean booth at room temperature and humidity.

During the sliding tests the frictional behavior of the collagen coating specimens was monitored in real time through a data acquisition system. Each test was repeated 3~5 times and an average friction coefficient was obtained for each experimental condition. After the sliding tests, the wear behavior of the collagen coatings was analyzed by measuring the wear scar formed on the surface due to abrasion. Wear scar measurement was done through analysis of images obtained from the SEM and the confocal microscope. The self-healing effect of the collagen coatings was tested by introducing water on the wear scar formed on the specimen due to abrasion during the sliding test. The degree of the self-healing was assessed by measuring the difference in the wear scar geometric profile before and after the introduction of water. In order to confirm the effect of temperature on the self-healing behavior of the collagen coatings, wear and healing tests were performed inside an oven for the high temperature (50 °C) test and inside a refrigerator for the low temperature (2 °C) test. Wear scars were generated on the specimen surface by using a reciprocating type of a tribotester that could fit inside the oven and the refrigerator. Wear tests were performed by sliding the collagen coating specimens against a ZrO_2_ ball with a diameter of 1 mm under an applied normal load of 29.4 mN for 100 cycles. The sliding stroke and speed were set to be 2 mm and 4 mm/s (1 Hz), respectively. After the wear test, the collagen coating specimens were treated with water at the given temperature through water treatment. The effectiveness of self-healing was assessed by observing the wear scar using confocal microscope before and after water treatment.

## Additional Information

**How to cite this article**: Kim, C.-L. and Kim, D.-E. Self-healing Characteristics of Collagen Coatings with Respect to Surface Abrasion. *Sci. Rep*. **6**, 20563; doi: 10.1038/srep20563 (2016).

## Figures and Tables

**Table 1:** XRD data and grain sizes of collagen coating before water treatment.

Peak	1	2	3	4	5	6	7	8
2θ,°	27.3°	28.3°	31.7°	45.4°	56.4°	66.2°	75.3°	84.0°
β_L_	0.00176	0.00182	0.00179	0.00172	0.00174	0.00193	0.00175	0.00192
D, nm	90	87	89	97	101	95	111	108

The typical broad diffraction pattern for collagen is found at 2θ = 24°. 8 peaks with high intensity were detected on the collagen coating before water treatment. Note that λ is the wavelength (0.154 nm), θ is the position of the diffraction peak, β_L_ is the full width at half maximum (FWHM), D is the grain size (nm) calculated by Equation (1).

**Table 2:** Mechanical properties and surface roughness of the collagen coating specimen before and after water treatment.

Collagen coating	Hardness (MPa)	Elastic modulus (MPa)	Stiffness (mN/μm)	Roughness (μm)
Before water treatment	0.2	15	1.5	2.4
After water treatment	1.4	66	7.6	0.2

Hardness, elastic modulus and stiffness were calculated from the indentation measurement data. The surface roughness of collagen coating was measured by the 3D profiler.

**Table 3:** Experimental conditions for indentation measurements and friction and wear tests.

Indentation test		Friction and wear test	
Loading force	Measurement value (mN)	Normal load	9.81 mN
Indentation depth	~3 μm	Sliding stroke	2 mm
Loading speed	0.1 μm/s	Sliding distance	400 mm
Unloading speed	0.1 μm/s	Sliding speed	4 mm/s
Tip material	Stainless steel (SUS 304)	Tip material	ZrO_2_ ball
Ball tip diameter	1 mm	Ball tip diameter	1 mm
